# Exploring the Therapeutic Mechanism of Tingli Dazao Xiefei Decoction on Heart Failure Based on Network Pharmacology and Experimental Study

**DOI:** 10.1155/2021/6645878

**Published:** 2021-11-25

**Authors:** Dan-Dan Zhao, Xiao-Qing Zhang, Tao Yang, Qian Liu, Zhen-Zhen Lan, Xiao-Li Yang, Hui-Yan Qu, Hua Zhou

**Affiliations:** ^1^Cardiovascular Research Institute, Shuguang Hospital Affiliated to Shanghai University of Traditional Chinese Medicine, Shanghai 201203, China; ^2^Cardiovascular Department of Traditional Chinese Medicine, Shuguang Hospital Affiliated to Shanghai University of Traditional Chinese Medicine, Shanghai 201203, China

## Abstract

**Background:**

Tingli Dazao Xiefei decoction (TDXD) has been shown to have a therapeutic effect on heart failure (HF). Nevertheless, its molecular mechanism for treating HF is still unclear.

**Materials and Methods:**

TDXD and HF targets were collected from the databases, and protein-protein interaction (PPI) analysis and enrichment analysis were performed on the overlapping targets. Then, AutoDock was employed for molecular docking. Finally, we used the left anterior descending coronary artery (LAD) ligation to induce HF model rats for in vivo experiments and verified the effect and mechanism of TDXD on HF.

**Results:**

Network pharmacological analysis showed that the main active components of TDXD in treating HF were quercetin, kaempferol, beta-carotene, isorhamnetin, and beta-sitosterol, and the core targets were IL-6, VEGFA, TNF, AKT1, and MAPK1. Multiple gene functions and signaling pathways were obtained by enrichment analysis, among which inflammation-related, PI3K/Akt, and MAPK signaling pathways were closely related to HF. Furthermore, the molecular docking results showed that the core targets had good binding ability with the main active components. Animal experiments showed that TDXD could effectively improve left ventricular ejection fraction (EF) and left ventricular fractional shortening (FS), decrease left ventricular internal diastolic diameter (LVIDd) and left ventricular internal systolic diameter (LVIDs), reduce the area of myocardial fibrosis, and decrease serum BNP, LDH, CK-MB, IL-6, IL-1*β*, and TNF-*α* levels in HF rats. Meanwhile, TDXD could upregulate the expression of Bcl-2, downregulate the expression of Bax, and reduce cardiomyocyte apoptosis. At the same time, it was verified that TDXD could significantly decrease the expression of PI3K, P-Akt, and P-MAPK. Captopril showed similar effects.

**Conclusions:**

Combining network pharmacological analysis and experimental validation, this study verified that TDXD could improve cardiac function and protect against cardiac injury by inhibiting the activation of PI3K/Akt and MAPK signaling pathways.

## 1. Introduction

Heart failure (HF) is the terminal stage of most cardiovascular diseases with high morbidity and mortality [1]. With the increase in the aging population, the incidence of HF has also been growing year by year [2]. The latest research data have shown that the prevalence of HF in Chinese adults is about 0.9%, and the number of HF patients has reached 4.5 million, thus becoming a major public health problem [3]. Despite great advances being made in the treatment of HF, the 5-year survival rate of HF is still comparable to that of malignant tumors [4]. HF is the dominant disease in traditional Chinese medicine (TCM) prevention and treatment. TCM syndrome differentiation and treatment of HF can not only play the role of multitarget and multilevel intervention but also has the advantages of fewer side effects, higher safety, and better effectiveness [5, 6].

Tingli Dazao Xiefei decoction (TDXD) originated from Zhang Zhongjing's “Synopsis of Golden Chamber” and has been widely used in clinical practice for more than 1000 years. TDXD, composed of Tinglizi (Semen Lepidii) and Dazao (Jujube), is a classic prescription for the treatment of HF with a definite curative effect [5]. Recent pharmacological studies have shown that Tinglizi can be diuretic, protect myocardial cells, enhance myocardial contractility, and inhibit ventricular remodeling [7, 8]. Dazao has antioxidant, anti-inflammatory, and hypolipidemic effects [9]. Animal studies confirmed that TDXD could inhibit the course of myocardial fibrosis and delay the progress of HF [10]. However, the active ingredients and specific molecular mechanisms of TDXD in the treatment of HF are still unknown.

Network pharmacology integrates systems biology and computer technology to construct the multidimensional “components-targets-pathways” network to reveal the pharmacological effects of TCM and the molecular mechanism of disease treatment, which is the research hotspot of TCM [11]. The study used network pharmacology to predict the molecular mechanism of TDXD in the treatment of HF and further verified through in vivo experiments, which will contribute to the clinical application of TDXD.

## 2. Materials and Methods

### 2.1. Chemicals and Reagents

TDXD dry powder was provided by the Chinese Pharmacy of Shuguang Hospital Affiliated to Shanghai University of Traditional Chinese Medicine. Captopril tablets (25 mg/tablet) were purchased from Beijing Jingfeng Pharmaceutical Group Co., Ltd. (Beijing, China). Hematoxylin-eosin dye (G1003), Masson dye (G1006), and TUNEL kit (G1501) were purchased from Wuhan Servicebio Technology Co., Ltd. (Wuhan, China). BNP ELISA kit (BPE30445), IL-6 ELISA kit (BPE30646), IL-1*β* ELISA kit (BPE30419), and TNF-*α* ELISA kit (BPE30635) were purchased from Lengton Bioscience Co., Ltd. (Shanghai, China). LDH assay kit (A020-2-2) was purchased from Nanjing Jiancheng Institute of Bioengineering (Nanjing, China). CK-MB assay kit (FSEA3390) was from Shanghai Fushen Biotechnology Co., Ltd. (Shanghai, China). BCA Protein Assay Kit (P0012S), protease and phosphatase inhibitor cocktail for general use (P1045), RIPA Lysis Buffer (P0013C), SDS-PAGE Gel Quick Preparation Kit (P0012AC), Bovine Serum Albumin (ST023), and stripping buffer (P0025) were purchased from Beyotime Biotechnology (Shanghai, China). Antibodies against PI3K (4249), Akt (4691), P-Akt (4060), MAPK (4695), P-MAPK (4370), GAPDH (5174), and rabbit IgG horseradish conjugate secondary antibody (7074) were from Cell Signaling Technology, Inc. (Boston, USA). Antibody against Bax was from Proteintech Group, Inc. (Wuhan, China). Antibody against Bcl-2 (40639) was from Signalway Antibody LLC, Inc. (Maryland, USA).

### 2.2. Screening of Active Compounds and Gene Targets of TDXD

The active components of Tinglizi and Dazao were collected from the Traditional Chinese Medicine Systems Pharmacology Database and Analysis Platform (TCMSP, http://lsp.nwu.edu.cn/tcmsp.php) [12]. The screening criteria for this study were oral bioavailability (OB) ≥30% and drug-likeness (DL) ≥0.18 [13]. Protein targets related to the active components were obtained from the TCMSP and entered into UniProt (http://www.uniprot.org/) [14]. The species was limited to “Homo sapiens,” so that the protein targets were converted to the corresponding gene symbols.

### 2.3. Screening of HF-Related Targets

GeneCards (https://www.genecards.org/) [15], Therapeutic Target Database (TTD, http://systemsdock.unit.osit.jp/iddp/home/index) [16], Online Mendelian Inheritance in Man (OMIM, http://omim.org/) [17], and DrugBank database (https://www.drugbank.ca) [18] were searched using “heart failure” as the keyword to collect HF-related targets. Then, the retrieval results from the four databases were merged, and the potential therapeutic targets of HF were obtained after deleting the duplicate targets.

### 2.4. Construction of Active Compounds-Disease Targets Network

The overlapping targets of TDXD and HF were acquired by the Venny 2.1 online tool (http://bioinfogp.cnb.csic.es/tools/venny/index.html). The active components in TDXD that failed to act on HF-related targets were eliminated. Next, the remaining components and overlapping targets were imported into Cytoscape 3.7.2 to construct the network of “active components-disease targets” [19]. The network analyzer plugin cytoNCA in the Cytoscape 3.7.2 was used for network topology analysis [20].

### 2.5. PPI Network Construction

The overlapping targets of TDXD and HF were inputted into the STRING 11.0 database (https://string-db.org/) [21]. The organism was set to “Homo sapiens,” and the minimum required interaction score was set to “highest confidence (0.9).” The disconnected nodes in the network were hidden to obtain PPI data. Subsequently, these data were uploaded into Cytoscape 3.7.2 for visualization and drew the PPI network. The topology analysis of the network was also carried out to acquire the core targets with the higher-ranking degree value.

### 2.6. Functional and Pathway Enrichment Analysis

The Gene Ontology (GO) functional and Kyoto Encyclopedia of Genes and Genomes (KEGG) pathway analysis of the overlapping targets was performed using the Database for Annotation, Visualization, and Integrated Discovery (DAVID v6.8, https://david.ncifcrf.gov/) [22]. With *P* < 0.05 as the screening criteria, according to the *P* value and the number of enrichment genes, the top 20 pathways were selected for visualization. Pathways related to HF were obtained by combining the literature search and the first 20 KEGG pathways. Cytoscape 3.7.2 was then used to construct the “compounds -targets-pathways” network diagram.

### 2.7. Molecular Docking

AutoDock 4.2.6 and PyMOL software were used to dock the core targets with the main active ingredients of TDXD [23]. The crystal structures of the core targets were downloaded from the protein data bank (PDB, https://www.rcsb.org) [24] and saved in pdb format as the receptors in molecular docking. PyMOL removed the original ligand structure in the receptor. The 3D structures (mol2 format) of active ingredients were obtained from the TCMSP database and converted into pdb format by PyMOL as the ligands in molecular docking. The receptors and ligands were processed by AutoDockTools (version 1.5.6) and stored in pdbqt format. After that, the processed receptors and ligands were molecularly docked through AutoDock. The parameter of the docking site was set to include the receptor's active pocket, and the search parameter was chosen as “Genetic Algorithm.” The lower the binding energy is, the better the binding affinity between the receptor and the ligand [25]. Finally, the conformation of the best binding energy was visualized by PyMOL.

### 2.8. Animal Experiments

All animal experiment protocols involved in this study were approved by the Animal Protection Committee of Shanghai University of Traditional Chinese Medicine with the approval number “PZSHUTCM210903018” and the experiments were performed in compliance with the guide for “Animal Research: Reporting of In Vivo Experiments (ARRIVE).” Forty SPF Wistar male rats (200 ± 10 g) were purchased from Beijing Weitong Lihua Co., Ltd. (Beijing, China) and raised in the Experimental Animal Center of Shanghai University of TCM. After 7 days of acclimation to the laboratory, 40 rats were randomized into four groups (*n* = 10): the sham group, the model group, the TDXD group, and the captopril group. Except for the sham group, the other 30 rats underwent ligation of the left anterior descending coronary artery (LAD) to replicate the myocardial infarction (MI) model as previously described [26]. Briefly, after anesthesia, the rats were connected to a rodent ventilator, left thoracotomy and subsequent pericardiotomy were performed to expose the heart, and the LAD was permanently ligated with 6/0 silk suture to induce MI. Through the paleness and cyanosis of the anterior wall of the left ventricle, the immediate elevation of the electrocardiogram confirmed the success of myocardial infarction modeling [27]. The sham group was only threaded under LAD without ligation. After the operation, rats were given penicillin (4 × 10^5^ units/day, intramuscular injection) for 3 days. During the whole experimental period, the total mortality of rats that underwent induction of HF was 30%. Most deaths occurred on the day of or the day after the surgery, probably due to severe arrhythmia or acute pump failure.

Administration dosages of TDXD (0.675 g/kg·d) and captopril (4.5 mg/kg·d) in rats were determined according to the equivalent patient dose [28]. For drug preparation, TDXD dry powder and captopril were dissolved in distilled water in doses of 67.5 mg/mL and 0.45 mg/mL, respectively. Then, according to the weight, the rat gavage volume was 1 mL/100 g. Rats in sham and model groups received the same volume of saline. One day after the surgery, rats were treated with a daily gastric solution using an oral gavage needle for 6 weeks.

### 2.9. Echocardiography

After the last administration, the rats were fasted for 12 hours and anesthetized with isoflurane inhalation [27]. The Vevo 2100 imaging system (Visual sonic Inc., Toronto, ON, Canada) was used to evaluate the cardiac function of the rats. Two-dimensional images of M-mode ultrasound were captured from the short axis view. Cardiac function indices include left ventricular ejection fraction (EF), left ventricular fractional shortening (FS), left ventricular internal diastolic diameter (LVIDd), and left ventricular internal systolic diameter (LVID).

### 2.10. Histological Analysis

The heart tissues of rats in each group were fixed in 4% paraformaldehyde for more than 24 hours, then dehydrated, and embedded in paraffin. Next, 5 *μ*m slices were cut for hematoxylin-eosin (H&E) and Masson staining, respectively, to observe the pathological damage and fibrosis of myocardial tissue [29]. ImageJ software (NIH, Bethesda, MD, USA) was used for quantitative analysis.

### 2.11. Terminal Deoxynucleotidyl Transferase-Mediated dUTP Nick End-Labeling (TUNEL) Assay

First, the slices were dewaxed with water, and antigenic repair was performed with protease K. After room temperature equilibrium, TUNEL color reaction solution was added and incubated for 2 hours at 37°C. Then, DAPI was added to the slices and incubated for 10 min. Finally, the slices were sealed with an antifluorescence quenching agent and observed under a fluorescence microscope. ImageJ software was used to count the number of TUNEL-positive cardiomyocyte nuclei. TUNEL-positive (%) = apoptotic nuclei number/total nuclei number × 100% [30].

### 2.12. ELISA

After the abdominal aorta blood was collected, it was left standing at room temperature for 2 h and centrifuged at 3000 rpm/min at 4°C for 15 min, and the supernatant was taken. The serum levels of BNP, LDH, CK-MB, IL-6, IL-1*β*, and TNF-*α* were detected according to ELISA kit instructions.

### 2.13. Western Blotting Assay

The myocardial tissue in the marginal zone of left ventricular infarction was homogenized, and the total protein concentration was determined by the BCA protein detection kit. The equivalent protein samples were separated by 10%–12% SDS polyacrylamide gel electrophoresis and transferred to a PVDF membrane, followed by western blotting as previously described [31]. The chemiluminescence system (Tanon Science & Technology Co., Shanghai, China) was used to obtain protein bands, and ImageJ software was used for quantitative analysis.

### 2.14. Statistical Analysis

SPSS 23.0 software (IBM Corp., Armonk, NY, USA) was used for statistical analysis, and data were expressed as mean ± standard deviation. One-way analysis of variance (ANOVA) followed by Bonferroni or Dunnett's T3 post hoc test was used for multiple comparisons, and value of *P* < 0.05 was considered to be statistically significant.

## 3. Results

### 3.1. Network Pharmacology Analysis of TDXD in the Treatment of HF

#### 3.1.1. Active Compounds and Targets of TDXD

With OB ≥30% and DL ≥0.18 as screening conditions, 39 active ingredients of TDXD were screened, including 12 compounds from Tinglizi and 29 compounds from Dazao. Quercetin and beta-sitosterol were the common active ingredients (Supplementary Materials, Table S1). In all, 221 gene targets were obtained after eliminating duplications in 39 compounds (Supplementary Materials, Table S2).

#### 3.1.2. HF-Related Targets and Overlapping Targets of TDXD and HF

**Figure 1:**
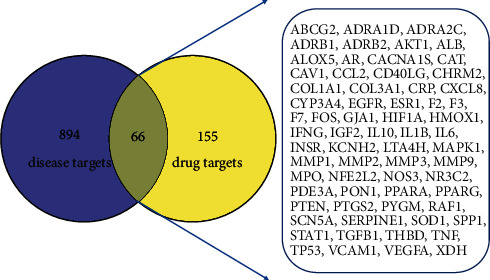
Venn diagram of TDXD targets and HF targets.

Gene targets of HF were obtained through GeneCards, taking the top 500 targets with the highest correlation according to the “Relevance score,” and then merging them with the results retrieved from TTD, OMIM, and DrugBank. After deleting duplicates values, 960 HF-related targets were identified. The Venn diagram was used to take the intersection of drug targets and disease targets, and 66 overlapped targets were screened (Figure 1). The overlapping targets were the potential targets for TDXD in treating HF.

#### 3.1.3. Active Compounds-Disease Targets Network

**Figure 2:**
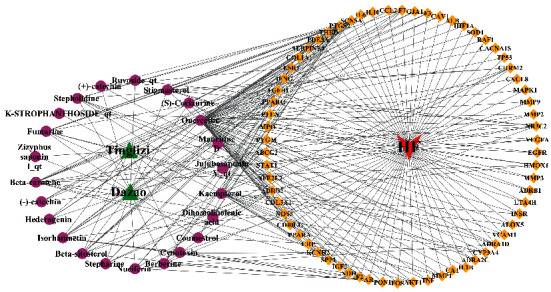
Network of active compounds-disease targets. Green regular triangle nodes represent the Tinglizi and Dazao. Purple round nodes represent the active components of TDXD. Red inverted triangle node represents HF. Yellow rhombic nodes represent the gene targets.

The 66 overlapping targets correspond to 23 active ingredients. In Cytoscape 3.7.2, the overlapping targets and corresponding active ingredients were introduced to construct the “active compounds-disease targets” network (Figure 2). This network included 92 nodes and 258 edges. The topological analysis showed that the top 5 active ingredients in degree value were quercetin, kaempferol, beta-carotene, isorhamnetin, and beta-sitosterol (Supplementary Materials, Table S3). The compounds with the higher degree value may be the main material basis of TDXD in the treatment of HF.

#### 3.1.4. PPI Network

**Figure 3:**
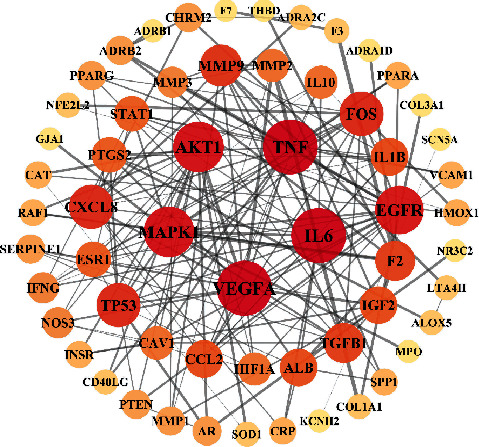
The PPI network of overlapping targets. Circular nodes represent the gene targets; the darker the node color and the larger the graph, the greater the degree value. Black lines represent the interaction between nodes, and the thicker the line, the closer the connection.

The 66 overlapping targets were uploaded to STRING to obtain the interaction diagram, after which Cytoscape 3.7.2 was used to visualize and construct the PPI network (Figure 3). A total of 59 nodes and 160 edges were involved in the PPI network. IL-6, VEGFA, TNF, AKT1, and MAPK1 were the top 5 targets of degree, showing that these targets were the core targets of TDXD in the treatment of HF. Topological parameters of the top 10 targets in the PPI network are shown in Supplementary Materials, Table S4.

#### 3.1.5. GO Functional and KEGG Pathways Enrichment Analysis

**Figure 4:**
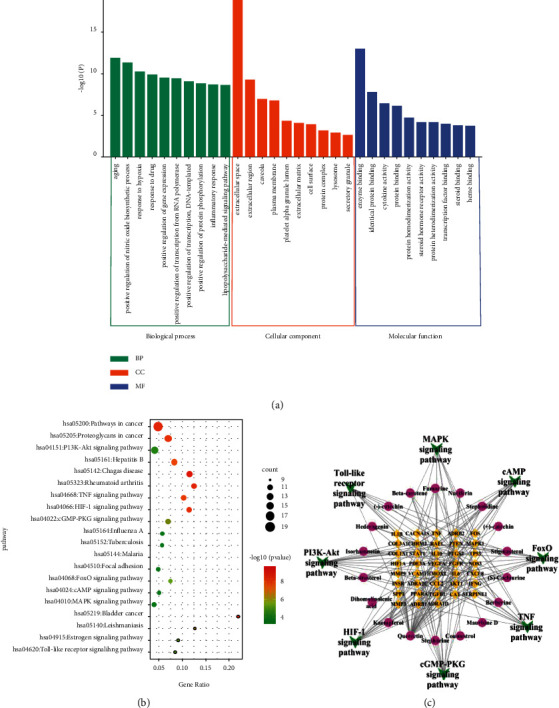
Enrichment analysis of TDXD against HF: (a) GO analysis of overlapping targets. The figure lists the top 10 GO pathways in each category. (b) Top 20 pathways of KEGG enrichment analysis. Bubble size represents the number of genes in this pathway, and the color reflects the −log10 (*P* value). (c) Compounds-targets-pathways network. Purple round nodes represent the active components of TDXD. Yellow rhombic nodes represent the gene targets. Green inverted triangle nodes represent the signaling pathways.

GO function analysis included three parts: biological process (BP), cell component (CC), and molecular function (MF). There were 294 BP entries, including aging, positive regulation of nitric oxide biosynthetic process, response to hypoxia, response to the drug, and so on; 30 CC entries, including extracellular space, extracellular region, plasma membrane, lysosome, and others; and 47 MF entries, including enzyme binding, protein binding, cytokine activity, and protein homodimerization activity. According to the sequencing of *P* values, the top 10 GO pathways in each category were selected to plot the bar chart (Figure 4(a)). A total of 87 pathways were obtained by KEGG enrichment analysis, which were sorted according to the number of enriched genes. The top 20 pathways are shown in Figure 4(b).

Based on the results of the literature search and KEGG pathway enrichment, there were 8 pathways related to HF in the first 20 pathways (Supplementary Materials, Table S5). The key pathways were PI3K/Akt, MAPK, TNF, HIF-1, and the Toll-like receptor signaling pathway, which mainly regulate complex biological metabolic processes such as cell proliferation, apoptosis, and inflammation. The targets enriched in these pathways and the active components corresponding to the targets were collected and uploaded to Cytoscape 3.7.2 to construct the “components-targets-pathways” network diagram (Figure 4(c)), which intuitively illustrates the characteristics and advantages of multicomponent, multitarget, and multipathway in HF treatment with TDXD.

#### 3.1.6. Molecular Docking

**Figure 5:**
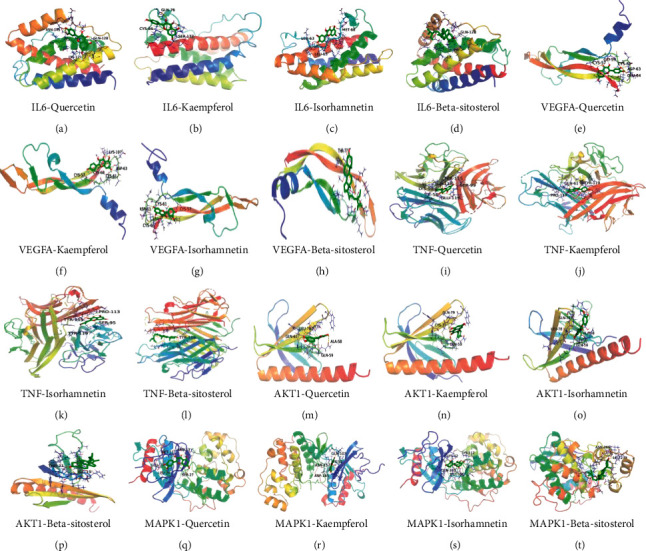
Molecular docking structure diagram. (a) IL-6-quercetin, (b) IL-6-kaempferol, (c) IL-6-isorhamnetin, (d) IL-6-beta-sitosterol, (e) VEGFA-quercetin, (f) VEGFA-kaempferol, (g) VEGFA-isorhamnetin, (h) VEGFA-beta-sitosterol, (i) TNF-quercetin, (j) TNF-kaempferol, (k) TNF-isorhamnetin, (l) TNF-beta-sitosterol, (m) AKT1-quercetin, (n) AKT1-kaempferol, (o) AKT1-isorhamnetin, (p) AKT1-beta-sitosterol, (q) MAPK1-quercetin, (r) MAPK1-kaempferol, (s) MAPK1-isorhamnetin, and (t) MAPK1-beta-sitosterol.

The top 5 core targets (IL-6, VEGFA, TNF, AKT1, and MAPK1) in the PPI network were docked with the main active components (quercetin, kaempferol, beta-carotene, isorhamnetin, and beta-sitosterol) of TDXD in treating HF and the positive drug (captopril) for HF. Because beta-carotene has no free hydrogen bond, it cannot stably bind to the target through hydrogen bond, so it is eliminated. It is generally believed that when the binding energy of ligand and receptor is <−4 kcal/mol, there is potential binding activity between them; binding energy <−5 kcal/mol suggests a significant binding ability between them [32]. Our docking results showed that the binding energy of the receptor proteins and the active ingredients were all lower than −5 kcal/mol, indicating that the receptor proteins and the active ingredients were well bound, which further provides evidence support for TDXD treatment of HF (Table 1). The conformation with the best binding energy during docking was downloaded and visualized using PyMOL (Figure 5).

### 3.2. Animal Experimental Study

#### 3.2.1. TDXD Improved Cardiac Function in Rats with HF

**Figure 6:**
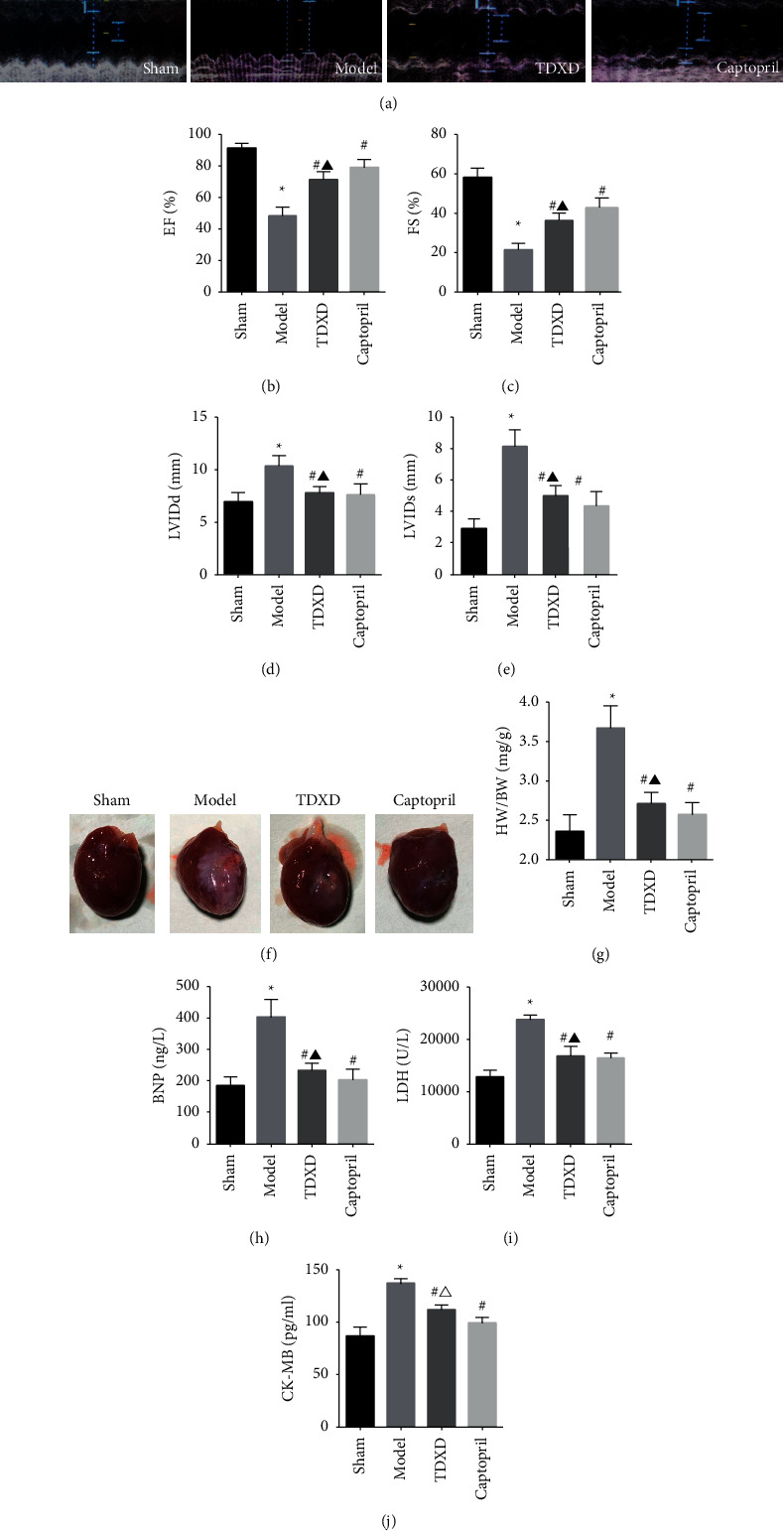
TDXD improved cardiac function in rats with HF. (a) Representative echocardiography images in each group, (b) EF%, (c) FS%, (d) LVIDd, and (e) LVIDs, *n* = 5. (f) Representative pictures of whole heart in each group, (g) HW/BW, *n* = 5. (h–j) Serum BNP, LDH, and CK-MB levels, *n* = 5. ^*∗*^*P* < 0.05 vs. sham group, ^#^*P* < 0.05 vs. model group, ^▲^*P* > 0.05 vs. captopril group, and ^Δ^*P* < 0.05 vs. captopril group.

As shown in Figures 6(a)–6(e), compared with the sham group, the EF and FS of the model group were reduced, and the LVIDd and LVIDs were increased, suggesting that the HF rat model was successfully established. While after treatment with TDXD and captopril, the levels of EF and FS were significantly upregulated, accompanied by the declined levels of LVIDd and LVIDs . In addition, the heart-to-body weight ratio (HW/BW) was also measured, as shown in Figures 6(f) and 6(g). The HW/BW ratio of the model group increased markedly, and TDXD could attenuate the HW/BW ratio to reduce the expansion of the ventricular cavity and myocardial hypertrophy. No significant differences in the above indexes between the TDXD group and the captopril group were noted.

Under normal circumstances, BNP, LDH, and CK-MB are located in the cytoplasm of cardiomyocytes. The increase of their levels in serum is usually considered as the diagnostic markers of HF and is positively correlated with the severity of HF [33]. The results showed that, compared with the sham group, the BNP, LDH, and CK-MB levels in the model group were significantly upregulated, while TDXD and captopril treatment reversed these changes. Moreover, captopril had a more significant effect on reducing serum CK-MB levels (Figures 6(h)–6(j)). The abovementioned results indicate that TDXD has a protective effect on heart function and structure.

#### 3.2.2. TDXD Reduced Myocardial Fibrosis in Rats with HF

**Figure 7:**
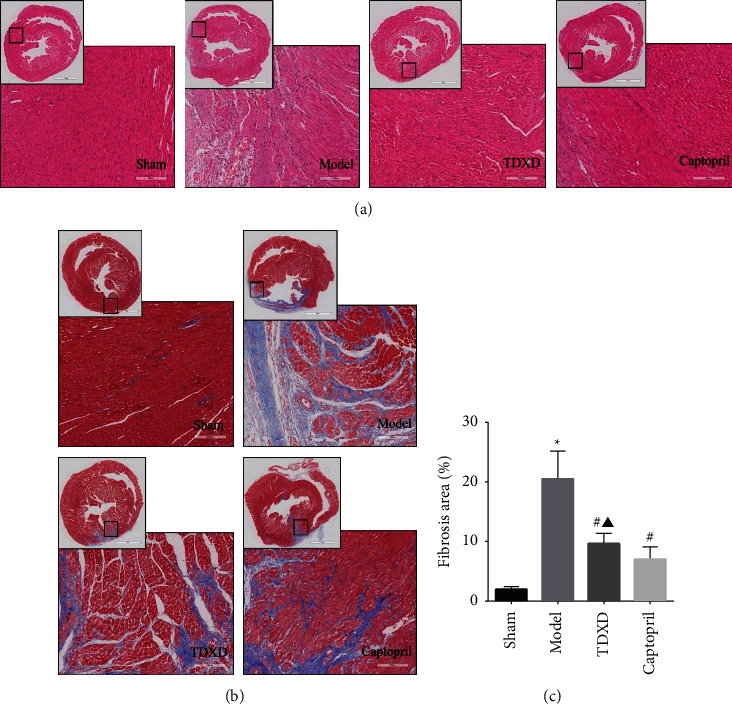
TDXD reduced myocardial fibrosis in rats with HF. (a) Representative images of H&E staining in different groups, *n* = 5. Scale bar: 3 mm/200 *μ*m. (b) Representative images of Masson staining in different groups, *n* = 5. Scale bar: 3 mm/200 *μ*m. (c) Quantitative analysis of the myocardial fibrosis area, *n* = 5. ^*∗*^ < 0.05 vs. sham group, ^#^*P* < 0.05 vs. model group, and ^▲^*P* > 0.05 vs. captopril group.

Ventricular remodeling caused by myocardial fibrosis is the main pathological mechanism of HF [34]. H&E and Masson staining were performed to determine whether TDXD could alleviate interstitial fibrosis in post-MI HF rats. The results showed that the cardiomyocytes in the sham group were normal in shape and orderly manner, without inflammatory infiltration and a very small amount of scattered collagen fiber tissue. In the model group, myocardial cells were degenerated, necrotic, and arranged disorderly, with a large number of inflammatory cells infiltration and collagen fibers deposition. Compared with the model group, myocardial tissue lesion, myocardial cell necrotic hypertrophy, and collagen fiber deposition were distinctly reduced in the TDXD group and the captopril group. There was no significant difference in myocardial fibrosis area between the TDXD group and the captopril group (Figures 7(a) and 7(c)).

#### 3.2.3. TDXD Inhibited Cardiomyocyte Apoptosis and Reduced the Level of Serum Inflammatory Cytokines

**Figure 8:**
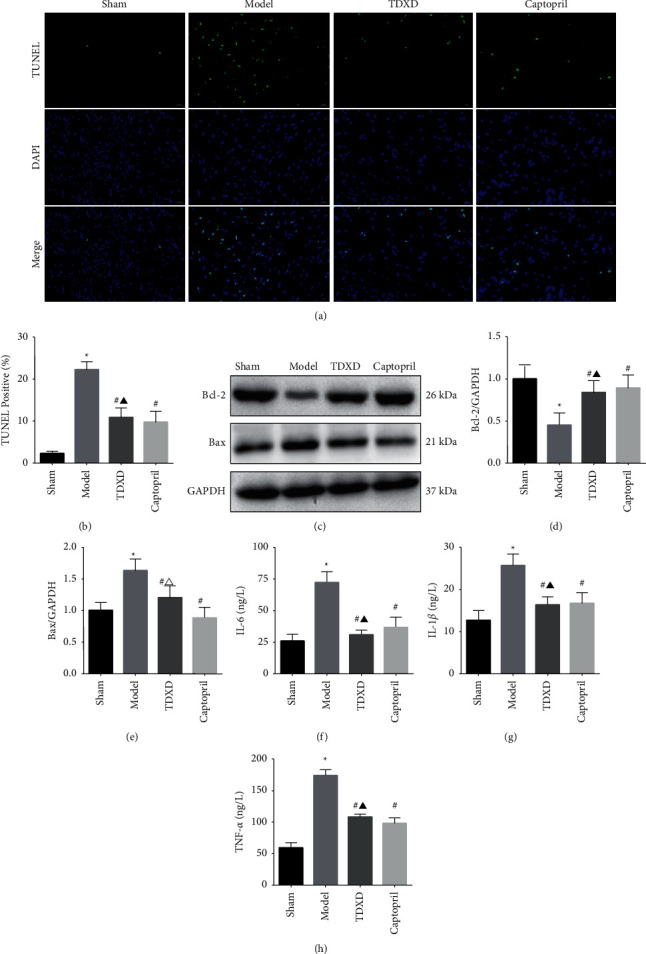
TDXD inhibited apoptosis and reduced the level of inflammatory cytokines. (a) Representative images of TUNEL staining in different groups, scale bar: 20 *μ*m. (b) Quantitative analysis of TUNEL-positive, *n* = 3. (c–e) Representative western blotting pictures and quantitative analysis of Bcl-2 and Bax, *n* = 5. (f–h) Serum IL-6, IL-1*β*, and TNF-*α* levels, *n* = 5. ^*∗*^*P* < 0.05 vs. sham group, ^#^*P* < 0.05 vs. model group, ^▲^*P* > 0.05 vs. captopril group, and ^Δ^*P* < 0.05 vs. captopril group.

Cardiomyocyte apoptosis is one of the important pathological mechanisms of myocardial injury, which is closely related to the severity of HF, inflammation, and myocardial fibrosis [35]. To identify whether TDXD can protect cardiomyocytes from apoptosis, TUNEL staining and western blotting were used to detect the level of apoptotic biomarkers. TUNEL results showed that the apoptotic rate of cardiomyocytes in the model group was dramatically higher than that in the sham group. However, TDXD and captopril treatment could notably reduce the apoptotic rate (Figures 8(a) and 8(b)). Western blotting further confirmed the antiapoptotic characteristic of TDXD. The results showed that, compared with the sham group, the expression of Bcl-2 was downregulated and the expression of Bax was upregulated in the model group, while TDXD and captopril could increase the expression of Bcl-2 and reduce the expression of Bax. In addition, the expression of Bax in the captopril group reduced more significantly (Figures 8(c)–8(e)).

Moderate regulation of the inflammatory response after MI is very important for the prognosis of HF. IL-6, IL-1*β*, and TNF-*α* are all inflammatory factors with multiple effects in the body, which are closely related to the occurrence and development of HF. The ELISA results showed that compared with the sham group, the serum levels of IL-6, IL-1*β*, and TNF-*α* in the model group increased significantly, suggesting a severe inflammatory response in HF. TDXD and captopril treatment reduced the levels of these inflammatory cytokines. Furthermore, compared with captopril, TDXD showed no significant difference in inhibiting IL-6, IL-1*β*, and TNF-*α* levels in HF rats (Figures 8(f)–8(h)).

#### 3.2.4. TDXD Inhibited the Activation of PI3K/AKT and MAPK Signaling Pathways

**Figure 9:**
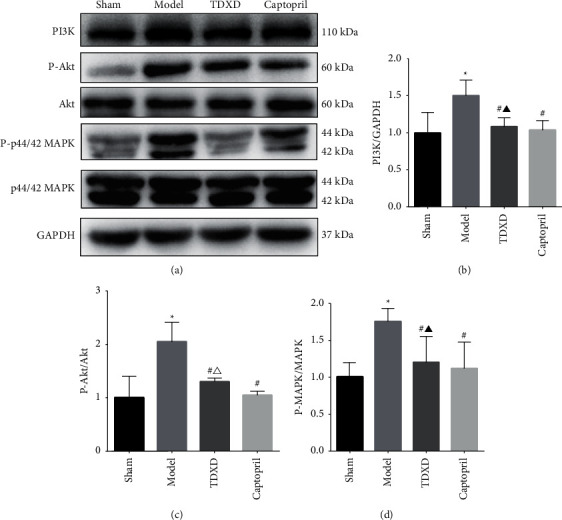
TDXD inhibited the activation of PI3K/AKT and MAPK signaling pathways. (a–d) Representative western blotting pictures and quantitative analysis of PI3K, p-Akt/AKT, and p-MAPK/MAPK, *n* = 5. ^∗^*P* < 0.05 vs. sham group, ^#^*P* < 0.05 vs. model group, ^▲^*P* > 0.05 vs. captopril group, and ^Δ^*P* < 0.05 vs. captopril group.

On the basis of network pharmacology analysis and literature research, in order to explore the underlying mechanism of TDXD in HF, PI3K/Akt, and MAPK signaling pathways were investigated. The PI3K/Akt signaling pathway can affect cardiac function through various mechanisms such as regulation of cardiomyocyte apoptosis, energy metabolism, oxidative stress, inflammatory response, and autophagy [36]. The MAPK signaling pathway can inhibit endoplasmic reticulum stress to regulate cardiomyocyte apoptosis. It can also regulate the expression of various inflammatory factors, such as IL-6 and TNF-*α*, so as to relieve the inflammatory response [37, 38]. Both of them play important roles in HF. According to the results, the expressions of PI3K, P-Akt, and P-MAPK in the model group were enhanced. TDXD and captopril could inhibit the activation of PI3K/Akt and MAPK pathways and reduce the expression levels of PI3K, P-Akt, and P-MAPK. Furthermore, the effect of captopril on reducing the expression of P-Akt was more significant (Figures 9(a) and 9(d)).

## 4. Discussion

HF has always been a hot and difficult problem in the field of cardiovascular research because of its high morbidity and mortality [39]. Recent studies have supported that TCM could treat diseases via acting on multiple targets, multiple signaling pathways, and multiple physiological functions, which have been clinically validated as a good means to treat HF [40]. This study revealed the material basis and potential mechanism of TDXD anti-HF through network pharmacology and conducted preliminary verification through in vivo experiments.

Network pharmacology can reveal the material basis of TCM's ability to treat diseases and provide a reference for follow-up research. The results showed that quercetin, kaempferol, beta-carotene, isorhamnetin, and beta-sitosterol were the most important compounds. Studies have found that quercetin can downregulate the TLR4/NF-*κ*B inflammatory pathway to inhibit the inflammatory response of cardiomyocytes and directly regulate the metabolism of NO in the heart to alleviate myocardial injury [41, 42]. Kaempferol has a cardioprotective role by regulating AMPK/Nrf2 and NF-*κ*B/MAPK pathways to prevent cardiac dysfunction and fibrosis [43]. Previous studies have confirmed that isorhamnetin can block the activation of the PI3K/Akt signaling pathway and reduce the process of cardiac hypertrophy and HF [44]. Beta-carotene has proved to have an obvious antioxidant effect and can reduce the oxidative stress level of tissues during HF to improve cardiac dysfunction [45]. Beta-sitosterol can reduce the area of myocardial infarction and cardiac cell apoptosis by modulating PPAR*γ*/NF-*κ*B signals to delay myocardial damage [46]. The abovementioned studies confirmed that the main active compounds have a cardioprotective effect mainly from regulating inflammation, antiapoptosis, and improving energy metabolism, which is consistent with the results of animal experiments in this study.

HF is associated with both local and systemic activation of inflammatory signaling cascades [47]. The inflammatory response of HF is characterized by the induction and activation of a wide range of pleiotropic cytokines and chemokines, which lead to the deterioration of cardiac remodeling and function [48]. Therefore, inhibiting the body's inflammatory response will be effective in the treatment of HF. In the analysis of core targets and KEGG enrichment pathways, IL-6, TNF, and Toll-like receptor signaling pathways were related to inflammation. IL-6, IL-1*β*, and TNF-*α* belong to the cytokine family, which are the key regulators of inflammation and injury. Their overexpression can contribute to the progression of HF by triggering apoptotic responses, perturbing calcium homeostasis, and damaging the function of endothelial cells and fibroblasts, among other ways [48]. Current studies have also supported that anti-inflammatory treatments for HF, such as IL-1*β* inhibitors canakinumab and gevokizumab, could reduce the hospitalization rate of HF and have a protective effect on HF patients [49, 50]. In this study, the results showed that TDXD could reduce serum IL-6, IL-1*β*, and TNF-*α* levels in HF rats, indicating that the cardioprotective effect of TDXD was related to the regulation of the body's inflammatory response.

PI3K/Akt is a classical signal transduction pathway, which can directly regulate the expression of Bcl-2/Bax and plays an important role in regulating cardiomyocyte survival, myocardial remodeling, and inflammation [51]. Upon receiving a stimulus, PI3K converts PIP2 to PIP3, which recruits Akt into the membrane and phosphorylates it for activation [52]. The phosphorylation level of Akt is one of the most important markers of the PI3K/Akt signal pathway. Studies have shown that overactivated P-Akt can lead to pathological hypertrophy of the heart, induce Bax gene expression, promote cardiomyocyte apoptosis, and accelerate the deterioration of cardiac function [53, 54]. MAPK1, also known as ERK, regulates Bcl-2 expression and activates the caspase cascade. Several studies have revealed that the MAPK/ERK signaling pathway regulates cell differentiation and apoptosis and participates in the process of myocardial remodeling [55]. Wohlschlaeger et al. confirmed that the levels of P-Akt and P-ERK in the myocardium of patients with HF increased. After treatment with the left ventricular assist device, the expression of P-Akt and P-ERK decreased significantly, and the cardiac function improved [56]. Chen et al. also found that inhibiting the phosphorylation of MAPK and PI3K/Akt pathways in vivo and in vitro can attenuate Ang II-induced cardiac hypertrophy [57]. The results of this study found that TDXD can significantly reduce the expression of P-Akt and P-MAPK in the cardiac tissue of rats with HF, suggesting that the role of TDXD in improving cardiac function, inhibiting cardiomyocyte apoptosis, and reducing the level of inflammatory cytokines is related to the regulation of PI3K/Akt and MAPK signaling pathways.

## 5. Conclusion

In summary, this study clarified the therapeutic effect and underlying mechanism of TDXD against HF through network pharmacology and in vivo experiments. It verified that TDXD inhibits the phosphorylation of PI3K/Akt and MAPK signaling pathways, thereby alleviating myocardial inflammation, fibrosis, apoptosis, and improving heart function in rats with HF. The results of this study provide a theoretical basis for further research and clinical application of TDXD.

## Figures and Tables

**Table 1:** The binding energy of molecular docking (kcal/mol).

	Quercetin	Kaempferol	Isorhamnetin	Beta-sitosterol	Captopril
IL-6 (1iL6)	−6.09	−6.17	−6.36	−8.72	−6.64
VEGFA (4KZN)	−5.34	−5.61	−5.86	−5.91	−4.96
TNF (6OP0)	−6.95	−8.15	−7.54	−8.36	−5.38
AKT1 (1UNP)	−6.27	−6.46	−6.49	−7.18	−6.33
MAPK1 (4FV8)	−6.68	−6.07	−6.68	−7.04	−6.74

## Data Availability

The data used to support the findings of this study are available from the corresponding author upon request.

## Supplementary Materials

Supplementary Materials Table S1: basic information on active ingredients in TDXD. Table S2: 221 gene targets of TDXD. Table S3: topological parameter of top 10 compounds in active compounds-disease targets network. Table S4: topological parameter of top 10 targets in PPI network. Table S5: parameter information of 8 pathways related to HF in the top 20 KEGG pathways.
