# Supplementary material for: Exploring the Therapeutic Mechanism of Tingli Dazao Xiefei Decoction on Heart Failure Based on Network Pharmacology and Experimental Study
Source: Evid Based Complement Alternat Med. 2021 Nov 25;2021:6645878. doi: 10.1155/2021/6645878 (PMC8639272; doi:10.1155/2021/6645878)
Supplement: Supplementary Materials — Table S1: basic information on active ingredients in TDXD. Table S2: 221 gene targets of TDXD. Table S3: topological parameter of top 10 compounds in active compounds-disease targets network. Table S4: topological parameter of top 10 targets in PPI network. Table S5: parameter information of 8 pathways related to HF in the top 20 KEGG pathways. [file 6645878.f1.doc]

T_ABLE_ S1: Basic information on active ingredients in TDXD

| Mol ID | Molecule name | OB (%) | DL | Herbs |
| --- | --- | --- | --- | --- |
| MOL000098 | Quercetin | 46.43 | 0.28 | Tinglizi, Dazao |
| MOL000358 | Beta-sitosterol | 36.91 | 0.75 | Tinglizi, Dazao |
| MOL002211 | 11,14-eicosadienoic acid | 39.99 | 0.20 | Tinglizi |
| MOL000296 | Hederagenin | 36.91 | 0.75 | Tinglizi |
| MOL000354 | Isorhamnetin | 49.6 | 0.31 | Tinglizi |
| MOL003905 | K-STROPHANTHOSIDE | 70.65 | 0.22 | Tinglizi |
| MOL003906 | K-STROPHANTHOSIDE qt | 30.80 | 0.78 | Tinglizi |
| MOL003907 | Erysimoside | 65.45 | 0.23 | Tinglizi |
| MOL003908 | Cynotoxin | 99.94 | 0.78 | Tinglizi |
| MOL003909 | Evobioside | 44.25 | 0.24 | Tinglizi |
| MOL003927 | Dihomolinolenic acid | 44.11 | 0.20 | Tinglizi |
| MOL000422 | Kaempferol | 41.88 | 0.24 | Tinglizi |
| MOL012921 | Stepharine | 31.55 | 0.33 | Dazao |
| MOL012940 | Spiradine A | 113.52 | 0.61 | Dazao |
| MOL012946 | Zizyphus saponin I qt | 32.69 | 0.62 | Dazao |
| MOL012961 | Jujuboside A qt | 36.67 | 0.62 | Dazao |
| MOL012976 | Coumestrol | 32.49 | 0.34 | Dazao |
| MOL012980 | Daechuine S6 | 46.48 | 0.79 | Dazao |
| MOL012981 | Daechuine S7 | 44.82 | 0.83 | Dazao |
| MOL012986 | Jujubasaponin V qt | 36.99 | 0.63 | Dazao |
| MOL012989 | Jujuboside C qt | 40.26 | 0.62 | Dazao |
| MOL012992 | Mauritine D | 89.13 | 0.45 | Dazao |
| MOL001454 | Berberine | 36.86 | 0.78 | Dazao |
| MOL001522 | (S)-Coclaurine | 42.35 | 0.24 | Dazao |
| MOL000211 | Mairin | 55.38 | 0.78 | Dazao |
| MOL000449 | Stigmasterol | 43.83 | 0.76 | Dazao |
| MOL003410 | Ziziphin qt | 66.95 | 0.62 | Dazao |
| MOL004350 | Ruvoside qt | 36.12 | 0.76 | Dazao |
| MOL000492 | (+)-catechin | 54.83 | 0.24 | Dazao |
| MOL005360 | Malkangunin | 57.71 | 0.63 | Dazao |
| MOL000627 | Stepholidine | 33.11 | 0.54 | Dazao |
| MOL007213 | Nuciferin | 34.43 | 0.40 | Dazao |
| MOL000783 | Protoporphyrin | 30.86 | 0.56 | Dazao |
| MOL000787 | Fumarine | 59.26 | 0.83 | Dazao |
| MOL008034 | 21302-79-4 | 73.52 | 0.77 | Dazao |
| MOL008647 | Moupinamide | 86.71 | 0.26 | Dazao |
| MOL002773 | Beta-carotene | 37.18 | 0.58 | Dazao |
| MOL000096 | (-)-catechin | 49.68 | 0.24 | Dazao |
| MOL013357 | (3S,6R,8S,9S,10R,13R,14S,17R)-7-[(1R, 4R)-4-ethyl-1,5-dimethy]-10,13-dimethyl-2,3,6,7,8,9,11,12,14,15,16,17-dodecahydr o-1H-cyclopenta[a]phenanthrene-3,6-diol | 34.37 | 0.78 | Dazao |

T_ABLE_ S2: Gene targets of TDXD

| **No.** | **Targets** | **No.** | **Targets** | **No.** | **Targets** | **No.** | **Targets** | **No.** | **Targets** |
| --- | --- | --- | --- | --- | --- | --- | --- | --- | --- |
| 1 | ABCG2 | 46 | CHEK2 | 91 | F2 | 136 | MAOA | 181 | PPP3CA |
| 2 | ACACA | 47 | CHRM1 | 92 | F3 | 137 | MAOB | 182 | PRKCA |
| 3 | ACHE | 48 | CHRM2 | 93 | F7 | 138 | MAP2 | 183 | PRKCB |
| 4 | ACP3 | 49 | CHRM3 | 94 | FASN | 139 | MAPK1 | 184 | PRSS1 |
| 5 | ADH1B | 50 | CHRM4 | 95 | FOS | 140 | MAPK14 | 185 | PSMD3 |
| 6 | ADH1C | 51 | CHRM5 | 96 | GABRA1 | 141 | MAPK8 | 186 | PTEN |
| 7 | ADRA1A | 52 | CHRNA2 | 97 | GABRA2 | 142 | MGAM | 187 | PTGER3 |
| 8 | ADRA1B | 53 | CHRNA7 | 98 | GABRA3 | 143 | MMP1 | 188 | PTGS1 |
| 9 | ADRA1D | 54 | CHUK | 99 | GABRA5 | 144 | MMP10 | 189 | PTGS2 |
| 10 | ADRA2A | 55 | CLDN4 | 100 | GABRA6 | 145 | MMP2 | 190 | PTPN1 |
| 11 | ADRA2B | 56 | COL1A1 | 101 | GJA1 | 146 | MMP3 | 191 | PYGM |
| 12 | ADRA2C | 57 | COL3A1 | 102 | GRIA2 | 147 | MMP9 | 192 | RAF1 |
| 13 | ADRB1 | 58 | CRP | 103 | GSK3B | 148 | MPO | 193 | RASA1 |
| 14 | ADRB2 | 59 | CTNNB1 | 104 | GSTM1 | 149 | MYC | 194 | RASSF1 |
| 15 | AHR | 60 | CTRB1 | 105 | GSTM2 | 150 | NCF1 | 195 | RB1 |
| 16 | AHSA1 | 61 | CTSD | 106 | GSTP1 | 151 | NCOA1 | 196 | RELA |
| 17 | AKR1B1 | 62 | CXCL10 | 107 | HAS2 | 152 | NCOA2 | 197 | RUNX1T1 |
| 18 | AKR1C3 | 63 | CXCL11 | 108 | HIF1A | 153 | NFE2L2 | 198 | RUNX2 |
| 19 | AKT1 | 64 | CXCL2 | 109 | HK2 | 154 | NFKBIA | 199 | RXRA |
| 20 | ALB | 65 | CXCL8 | 110 | HMOX1 | 155 | NKX3-1 | 200 | SCN5A |
| 21 | ALOX5 | 66 | CYP1A1 | 111 | HSF1 | 156 | NOS2 | 201 | SELE |
| 22 | AR | 67 | CYP1A2 | 112 | HSP90AB1 | 157 | NOS3 | 202 | SERPINE1 |
| 23 | BAX | 68 | CYP1B1 | 113 | HSPA5 | 158 | NPEPPS | 203 | SLC2A4 |
| 24 | BCL2 | 69 | CYP2B6 | 114 | HSPB1 | 159 | NQO1 | 204 | SLC6A2 |
| 25 | BCL2L1 | 70 | CYP3A4 | 115 | HTR2A | 160 | NR1I2 | 205 | SLC6A3 |
| 26 | BIRC5 | 71 | DCAF5 | 116 | HTR2C | 161 | NR1I3 | 206 | SLC6A4 |
| 27 | CA2 | 72 | DIO1 | 117 | HTR3A | 162 | NR3C2 | 207 | SLPI |
| 28 | CACNA1S | 73 | DPEP1 | 118 | ICAM1 | 163 | ODC1 | 208 | SOD1 |
| 29 | CAMKK | 74 | DPP4 | 119 | IFNG | 164 | OLR1 | 209 | SPP1 |
| 30 | CASP3 | 75 | DRD1 | 120 | IGF2 | 165 | OPRD1 | 210 | STAT1 |
| 31 | CASP7 | 76 | DRD2 | 121 | IGFBP3 | 166 | OPRM1 | 211 | SULT1E1 |
| 32 | CASP8 | 77 | DRD4 | 122 | IGHG1 | 167 | PARP1 | 212 | TGFB1 |
| 33 | CASP9 | 78 | DRD5 | 123 | IKBKB | 168 | PCOLCE | 213 | THBD |
| 34 | CAT | 79 | DUOX2 | 124 | IL10 | 169 | PDE10A | 214 | TNF |
| 35 | CAV1 | 80 | E2F1 | 125 | IL1A | 170 | PDE3A | 215 | TOP1 |
| 36 | CCL2 | 81 | E2F2 | 126 | IL1B | 171 | PGR | 216 | TOP2 |
| 37 | CCNA2 | 82 | EGF | 127 | IL2 | 172 | PIK3CG | 217 | TOP2A |
| 38 | CCNB1 | 83 | EGFR | 128 | IL6 | 173 | PIM1 | 218 | TP53 |
| 39 | CCND1 | 84 | EIF6 | 129 | INSR | 174 | PLAT | 219 | VCAM1 |
| 40 | CD40LG | 85 | ELK1 | 130 | IRF1 | 175 | PLAU | 220 | VEGFA |
| 41 | CDC37 | 86 | ERBB2 | 131 | JUN | 176 | PON1 | 221 | XDH |
| 42 | CDK1 | 87 | ERBB3 | 132 | KCNH2 | 177 | POR |  |  |
| 43 | CDK2 | 88 | ESR1 | 133 | KDR | 178 | PPARA |  |  |
| 44 | CDKN1A | 89 | ESR2 | 134 | KLF7 | 179 | PPARD |  |  |
| 45 | CHEK1 | 90 | F10 | 135 | LTA4H | 180 | PPARG |  |  |

T_ABLE_ S3: Topological parameter of top 10 compounds in active compounds-disease targets network

| Mol ID | compounds | Degree | Betweenness | Closeness | ASPL |
| --- | --- | --- | --- | --- | --- |
| MOL000098 | Quercetin | 58 | 0.304 | 0.659 | 1.516 |
| MOL000422 | Kaempferol | 19 | 0.026 | 0.414 | 2.418 |
| MOL002773 | Beta-carotene | 12 | 0.016 | 0.396 | 2.527 |
| MOL000354 | Isorhamnetin | 10 | 0.010 | 0.386 | 2.593 |
| MOL000358 | Beta-sitosterol | 10 | 0.012 | 0.396 | 2.527 |
| MOL000787 | Fumarine | 9 | 0.008 | 0.386 | 2.593 |
| MOL001454 | Berberine | 8 | 0.006 | 0.382 | 2.615 |
| MOL000449 | Stigmasterol | 8 | 0.011 | 0.389 | 2.571 |
| MOL000627 | Stepholidine | 8 | 0.005 | 0.382 | 2.615 |
| MOL001522 | (S)-Coclaurine | 7 | 0.004 | 0.379 | 2.637 |

T_ABLE_ S4: Topological parameter of top 10 targets in PPI network

| Targets | Degree | Betweenness | Closeness | ASPL |
| --- | --- | --- | --- | --- |
| IL6 | 16 | 0.109 | 0.505 | 1.982 |
| VEGFA | 16 | 0.163 | 0.519 | 1.929 |
| TNF | 16 | 0.100 | 0.483 | 2.071 |
| AKT1 | 14 | 0.134 | 0.459 | 2.179 |
| MAPK1 | 14 | 0.166 | 0.505 | 1.982 |
| EGFR | 13 | 0.113 | 0.496 | 2.018 |
| CXCL8 | 11 | 0.050 | 0.438 | 2.286 |
| TP53 | 11 | 0.064 | 0.475 | 2.107 |
| FOS | 11 | 0.081 | 0.467 | 2.143 |
| MMP9 | 10 | 0.075 | 0.434 | 2.304 |

T_ABLE_ S5: Parameter information of 8 pathways related to HF in the top 20 KEGG pathways

| Pathway | P value | Count | Genes |
| --- | --- | --- | --- |
| PI3K-Akt signaling pathway | 7.45E-06 | 14 | CHRM2, NOS3, INSR, PTEN, EGFR, VEGFA, COL1A1, COL3A1, IL6, SPP1, AKT1, MAPK1, RAF1, TP53 |
| HIF-1 signaling pathway | 8.73E-09 | 11 | IL6, IFNG, NOS3, INSR, SERPINE1, HMOX1, MAPK1, AKT1, HIF1A, EGFR, VEGFA |
| TNF signaling pathway | 2.52E-08 | 11 | IL6, VCAM1, IL1B, MMP3, MAPK1, CCL2, AKT1, FOS, PTGS2, TNF, MMP9 |
| cGMP-PKG signaling pathway | 1.01E-06 | 11 | NOS3, INSR, PDE3A, MAPK1, AKT1, ADRB1, ADRA1D, ADRB2, CACNA1S, RAF1, ADRA2C |
| FoxO signaling pathway | 2.27E-06 | 10 | IL10, IL6, TGFB1, INSR, CAT, PTEN, MAPK1, AKT1, RAF1, EGFR |
| cAMP signaling pathway | 5.36E-05 | 10 | CHRM2, PDE3A, MAPK1, AKT1, ADRB1, FOS, ADRB2, CACNA1S, RAF1, PPARA |
| MAPK signaling pathway | 0.0003 | 10 | TGFB1, IL1B, MAPK1, AKT1, FOS, CACNA1S, RAF1, TNF, TP53, EGFR |
| Toll-like receptor signaling pathway | 3.65E-06 | 9 | IL6, CXCL8, STAT1, IL1B, SPP1, MAPK1, AKT1, FOS, TNF |
